#  ‎ Anxiety Sensitivity Dimensions and Generalized Anxiety‏ ‏Severity: The ‎Mediating Role of Experiential Avoidance and Repetitive‏ ‏Negative Thinking‎ ‎

**Published:** 2016-07

**Authors:** Parvaneh Mohammadkhani, Abbas Pourshahbaz, Maryam Kami, Mahdi Mazidi, Imaneh‏ Abasi

**Affiliations:** Department of Clinical Psychology, School of Behavioral Sciences and Mental Health, University of Social Welfare and Rehabilitation Sciences, Tehran, Iran‎.

**Keywords:** *Anxiety Sensitivity*, *Experiential Avoidance*, *Generalized Anxiety Disorder*, *Repetitive Thinking*, ‎*Trans-Diagnostic Mechanisms*

## Abstract

**Objective:** Generalized anxiety disorder is one of the most common anxiety disorders in the general ‎population. Several studies suggest that anxiety sensitivity is a vulnerability factor in generalized ‎anxiety severity. However, some other studies suggest that negative repetitive thinking and ‎experiential avoidance as response factors can explain this relationship. Therefore, this study ‎aimed to investigate the mediating role of experiential avoidance and negative repetitive thinking ‎in the relationship between anxiety sensitivity and generalized anxiety severity.‎

**Method:** This was a cross-sectional and correlational study. A sample of 475 university students was ‎selected through stratified sampling method. The participants completed Anxiety Sensitivity ‎Inventory-3, Acceptance and Action Questionnaire-II, Perseverative Thinking Questionnaire, and ‎Generalized Anxiety Disorder 7-item Scale. Data were analyzed by Pearson correlation, multiple ‎regression analysis and path analysis.‎

**Results:** The results revealed a positive relationship between anxiety sensitivity, particularly cognitive ‎anxiety sensitivity, experiential avoidance, repetitive thinking and generalized anxiety severity. In ‎addition, findings showed that repetitive thinking, but not experiential avoidance, fully mediated ‎the relationship between cognitive anxiety sensitivity and generalized anxiety severity. α Level ‎was p<0.005.‎

**Conclusion:** Consistent with the trans-diagnostic hypothesis, anxiety sensitivity predicts generalized anxiety‏ ‏severity, but its effect is due to the generating repetitive negative thought.‎

Generalized anxiety disorder (GAD) is one of the most common disorders found in ‎clinical centers and the general population (1). The 12- month and lifetime prevalence ‎of this disorder has been estimated to be 3.6% to 4% and 9%, respectively. In the ‎Diagnostic and Statistical Manual of Mental Disorders, Fifth Edition (DSM-5), ‎generalized anxiety disorder is defined as “excessive worry and anxiety about different ‎events and activities, along with physical and cognitive symptoms that impair ‎function (2). Based on the taximetrics study, generalized anxiety disorder is better ‎represented as a dimensional construct rather than a categorical construct. Contrary to ‎the categorical model of DSM-5, some evidences do not support dichotomizing ‎individuals into disordered versus non-disordered groups and some suggest that any ‎diagnostic thresholds to identify GAD group are likely to be arbitrary. This allows the ‎investigators to study GAD as a continuum disorder whose severity varies in the general ‎population (3).‎

Many cross- sectional and longitudinal studies have been conducted on the risk factors ‎of this disorder and its severity. One of the factors found to play a role in generalized ‎anxiety severity is anxiety sensitivity (4, 5), meaning a fear of sensations and ‎consequences is associated with anxiety (6). ‎

Some studies have shown that levels of anxiety sensitivity are correlated with ‎generalized anxiety severity (5, 7), and that anxiety sensitivity is significantly higher in ‎people with generalized anxiety disorder than the controls, specially its cognitive factor ‎which involves items assessing one’s worries about mental capacity and performance ‎such as focusing and cognitive control (8, 9). Narimani et al. (2015) found that ‎generalized anxiety symptoms decrease by reducing anxiety sensitivity through applied ‎relaxation and cognitive-behavioral therapy, (10). ‎

Although several studies have shown the role of anxiety sensitivity in generalized ‎anxiety severity, the next step is to identify the mechanism, which relates the two ‎constructs. Some studies indicate that the cognitive factor of anxiety sensitivity relative ‎to other subscales has the strongest relationship with generalized anxiety. To explain ‎this, DSM-V stated that fear of lack of cognitive control is consistent with a cognitive ‎processing problem observed in generalized anxiety as uncontrollable and excessive ‎worry is the main cognitive characteristic of anxiety (1). On the other hand, several ‎studies have found that anxiety sensitivity predicts levels of worry in healthy and ‎anxious people (11). Therefore, it seems that considering worry as uncontrollable may ‎lead to an increase in fear and sensitivity to anxiety symptoms, followed by an increase ‎in anxiety (12). Consistent with this, Cox et al. (2001) found the mediating role of ‎rumination in the relationship between anxiety sensitivity and depression (13). Recent ‎studies indicate that worry and rumination are regarded – as cognitive processes ‎governing generalized anxiety and depression, and as parts of the latent variable, ‎repetitive thinking – and this may explain the comorbidity and common aspects ‎between the two disorders with respect to anxiety sensitivity. Therefore, anxiety ‎sensitivity through repetitive thinking may lead to severity of symptoms in generalized ‎anxiety.‎

Another factor related to generalized anxiety severity is experiential avoidance. ‎Experiential avoidance is a process including negative and excessive evaluations of ‎sensations, feelings, and unwanted private thoughts and a lack of interest in ‎experiencing these private events and voluntary efforts to control them or scape from ‎them. This factor as an evident aspect of most mental disorders, involves a general ‎pattern of intentional actions to eliminate undesirable mental states, which limits the ‎functioning of the person (14). ‎

Experiential avoidance and repetitive thinking, as trans-diagnostic response factors, can ‎explain the relationship between anxiety sensitivity and generalized anxiety. Recently, ‎some studies have shown that the relationship between anxiety sensitivity and some ‎disorders is through experiential avoidance (15). That is, some mechanisms are used to ‎regulate the emotions related to anxiety symptoms to minimize dealing with undesirable ‎experiences. The main reason that explains‏ ‏the relationship between experiential ‎avoidance and generalized anxiety is that people with severe generalized anxiety are ‎sensitive to their physical symptoms and internal emotions and are hyper‏-‏vigilant ‎toward real or imaginary unpredictable dangers. Therefore, they use avoidance or ‎control mechanisms (instead of acceptance) to manage their emotions (16). Moreover, ‎several studies confirm the relationship between vulnerability factors and response ‎factors that predict generalized anxiety severity. No study has integrated these ‎relationships in a model. Therefore, this study aimed to examine the mediating role of ‎experiential avoidance and repetitive thinking in the relationship between anxiety ‎sensitivity and generalized anxiety severity.‎‎

## Materials and Method

***Participants***

The study population included all students of two major universities in Tehran, who ‎were selected using stratified random sampling method (based on gender). After omitting ‎outliers and inappropriate questionnaires, a sample of 475 university students was selected. The ‎inclusion criterion was being above 18 years of age, and the exclusion criterion was a ‎report of drug use. ‎

***Instruments***

**Brief Measure of Generalized Anxiety Disorder (GAD-7):** This is a 7-item scale for ‎screening and assessing the severity of generalized anxiety, which was developed by ‎spritzer et al. (2006). The psychometric characteristics of its main edition are as ‎follows: Its internal consistency, using the Cronbach's alpha coefficient, and its two-‎week test-retest reliability coefficient was reported as 0.91 and 0.83, respectively. The ‎convergent validity of the scale, assessed by an examination of its correlations with the ‎Beck Depression Inventory (BDI) and the anxiety subscale of the SCL-90, was ‎calculated as 0.72 and 0.74, respectively (17). In Iran, Naeinian et al. (2012) found good ‎internal consistency (0.85). The convergent validity of the GAD-7, assessed by ‎measuring its correlations with Symptom Checklist 90 Revised (SCL-90-R) and state-trait ‎anxiety inventory (STAI), was calculated as 0.63 and 0.71 in student and clinical ‎samples (18).‎

**The Anxiety Sensitivity Index-3 (ASI-3): **Taylor et al. (2007) generated, and for the ‎first time, examined the psychometric properties of the third version of anxiety ‎sensitivity inventory. This version is an 18-item self-report questionnaire, assessing ‎psychological, cognitive and social aspects of anxiety sensitivity. It has three subscales ‎including cognitive, physical, and social. The psychometric characteristics of this scale ‎have been reported to be good (19). Allan et al. (2014), using the Cronbach's alpha ‎coefficient, reported the internal consistency of the scale as 0.92. The scale has a good ‎discriminant validity as well (20). In Iran, Kami et al. calculated its internal consistency, ‎using the Cronbach's alpha coefficient (0.85), and convergent validity, using calculating ‎of its correlation with acceptance and action questionnaire-II (AAQ-II), (0.5) (article in ‎press).‎

**The Acceptance and Action Questionnaire - II (AAQ-II):** Bond et al. developed this ‎questionnaire (2011). It assesses diversity, acceptance, experiential avoidance, and ‎psychological inflexibility. The psychometric characteristics of the main edition are as ‎follows: The mean alpha coefficient was .84, and the 3- and 12-month test-retest ‎reliability was calculated as .81 and .79, respectively. The scale has a good discriminant ‎validity (21). In Iran, Abbasi et al. (2013) reported the psychometric characteristics of ‎this questionnaire; an exploratory factor analysis revealed two factors: Avoiding ‎emotional experiences and control over life. The internal consistency and split-half ‎coefficient of the scale were good (0.89-0.71) (22).‎

**The Preservative Thinking Questionnaire (PTQ):** Ehring et al. developed this ‎questionnaire (2011) as an instrument for assessing repetitive thinking, independent ‎from contents (23). In a series of factor analyses, a model with a higher level factor ‎consisting of repetitive negative thoughts (RNT) and three lower level factors including ‎the main characteristics of RNT (repetitive, intruding, difficult to detach) perceiving the ‎uselessness of these thoughts and occupying the mental capacity of the person showed a ‎good fitting. The psychometric characteristics of its main edition are as follows: The ‎internal consistency, using a two-week test-retest reliability coefficient was reported as ‎‎0.69. The convergent validity of the scale, assessed by an examination of its ‎correlations with Penn State Worry Questionnaire (PSWQ) and the rumination scale of ‎the Response Style Questionnaire (RSQ), was calculated as 0.70 and 0.63, respectively. ‎In Iran, Kami et al. calculated its reliability, using test-retest examination (0.72), and ‎convergent validity, using calculating of its correlation with difficulty in emotion ‎regulation scale (DERS), 0.65 (article in press).‎

***Procedure***

After obtaining informed consent and explaining the aim and importance of the study to ‎the participants, the inclusion/exclusion criteria were examined. Then, participants ‎completed the printed sets of the questionnaires (including GAD-7, ASI-3, AAQ-2, and ‎PTQ). They asked questions about unclear items, and could write e-mails to receive the ‎study results. Then, questionnaires were examined to find incomplete or incorrect ‎answers. Five hundred fifty sets of questionnaires were distributed, and after removing ‎incomplete questionnaires and outliers, 475 sets of questionnaires were entered into the ‎statistical analysis.‎

***Statistical Analysis***

Statistical analysis was conducted using SPSS version 22. Pearson correlation ‎coefficient and multiple regression were used to examine the study hypotheses ‎‎ (α level<0.05).‎

## Results

***Descriptive Statistics***

The demographics of the sample are as follows: 256 men (53.9%) and 229 women (45.7%), with an average age of 22.53 and 3.13, respectively. Two of the participants ‎did not reveal their gender, and three did not mention their age.‎

The correlations, means and standard deviations of the study variables are displayed in ‎Table 1. Participants had a moderate level of generalized anxiety (M = 29.6). As it was ‎hypothesized, among the three factors of anxiety sensitivity, generalized anxiety had ‎the strangest correlation with the cognitive factor (r = 0.46, p<0.001). Generalized ‎anxiety had moderate to high correlations with repetitive thinking and experiential ‎avoidance (r = 0.54, p<0.001, r = 0.52, p<0.001, respectively). Repetitive thinking and ‎experiential avoidance are most correlated with the cognitive factor of anxiety ‎sensitivity (r = 0.47, p<0.001, r = 0.54, p<0.001, respectively).‎

**Table1:** Means, Standard Deviations of Anxiety Sensitivity Dimensions, Repetitive Thinking, Experiential Avoidance and Generalized Anxiety Severity, and Correlations Among Them(p<0.001)

		**1**	**2**	**3**	**4**	**5**	**6**	**7**	**M**	**SD**
1	**Anxiety sensitivity- Total**	1	0.88	0.85	0.83	0.49	0.53	0.46	20.54	12.8
2	**Anxiety sensitivity- Cognitive**		1	0.71	0.59	0.47	0.54	0.46	5.12	4.8
3	**Anxiety sensitivity- Physical**			1	0.52	0.37	0.42	0.43	6.13	4.8
4	**Anxiety sensitivity- Social**				1	0.41	0.41	0.31	9.28	5.2
5	**Repetitive thinking**					1	0.63	0.54	25.42	11.81
6	**Experiential avoidance**						1	0.52	32.9	10.01
7	**Generalized anxiety severity**							1	6.69	3.96

**Table2:** Summary of Regression Analysis for the Three Factors of Anxiety Sensitivity on Repetitive Thinking

		**B**	**Std. Error**	**Beta**	**R**	**R2**	**Adjusted R2**	**T**	**Sig**
1	**Constant**	15.83	0.95					16.52	0.0001
	**Anxiety sensitivity- Physical**	0.06	0.14	0.02				0.426	0.67
	**Anxiety sensitivity- Cognitive**	0.83	0.14	0.34				5.65	0.0001
	**Anxiety sensitivity- Social**	0.44	0.11	0.2	0.5	0.25	0.25	3.99	0.0001

**Table3:** Summary of Regression Analysis for the Three Factors of Anxiety Sensitivity on ‎Experiential Avoidance

		**B**	**Std. Error**	**Beta**	**R**	**R2**	**Adjusted R2**	**T**	**Sig**
1	**Constant**	24.46	0.78					31.36	0.0001
	**Anxiety sensitivity- Physical**	0.08	0.11	0.04				0.74	0.45
	**Anxiety sensitivity- Cognitive**	0.9	0.12	0.43				7.49	0.0001
	**Anxiety sensitivity- Social**	0.26	0.09	0.14	0.56	0.31	0.31	2.91	0.004

**Table4:** Summary of Regression Analysis for the Three Factors of Anxiety Sensitivity on Generalized Anxiety Severity

		**B**	**Std. Error**	**Beta**	**R**	**R2**	**Adjusted R2**	**T**	**Sig**
1	**Constant**	4.13	0.32					12.71	0.0001
	**Anxiety sensitivity- Physical**	0.17	0.04	0.21				3.64	0.0001
	**Anxiety sensitivity- Cognitive**	0.24	0.05	0.3				4.92	0.0001
	**Anxiety sensitivity- Social**	0.01	0.03	0.02	0.49	0.24	0.23	0.44	0.65

**Table5:** Summary of regression analysis for the 3 factors of anxiety sensitivity, experiential ‎avoidance, and repetitive thinking on generalized anxiety severity

		**B**	**Std. Error**	**Beta**	**R**	**R2**	**Adjusted R2**	**T**	**Sig**
1	**Constant**	0.22	0.5					0.45	0.65
	**Anxiety sensitivity- Physical**	0.16	0.04	0.19				3.76	0.0001
	**Anxiety sensitivity- Cognitive**	0.07	0.04	0.09				1.59	0.11
	**Anxiety sensitivity- Social**	-0.05	0.03	-0.07				-1.62	0.1
	**Repetitive thinking**	0.1	0.01	0.32				6.97	0.0001
	**Experiential avoidance**	0.08	0.01	0.22	0.63	0.4	0.39	4.6	0.0001

**Figure1:**
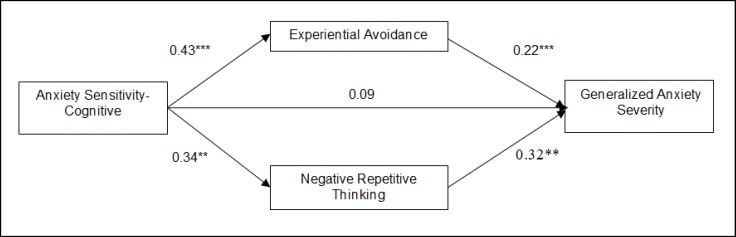
Path analysis model for mediating role of experiential avoidance and repetitive thinking in the relationship between anxiety sensitivity and generalized anxiety severity

**p <0.*001*

***p<0.0001

***Path Analysis***

The trans-diagnostic variables were associated with the severity of generalized anxiety. ‎In the next step of the path analysis, a multivariate regression analysis was used to ‎determine how much of the relationship between anxiety sensitivity and generalized ‎anxiety was explained by repetitive thinking and experiential avoidance. At first, the ‎three subscales of anxiety sensitivity served as predictive variables, and experiential ‎avoidance and repetitive thinking served as criterion variables in two distinct regression ‎analyses (Tables 2 and 3). As displayed in the tables, only the cognitive and social factors ‎were entered into the model for experiential avoidance and repetitive thinking. The ‎cognitive and social factors explain 0.25% of the variance of repetitive thinking, with ‎Beta coefficients of 0.34 and 0.2, respectively. Moreover, they explain 0.31% of the ‎variance of experiential avoidance, with Beta coefficients of 0.12 and 0.09, ‎respectively. The role of physical factor was not 

significant in repetitive thinking and ‎experiential avoidance.‎

In the next step, the role of the three anxiety sensitivity factors in the severity of ‎generalized anxiety was examined. The social factor did not significantly explain the ‎severity of generalized anxiety, but the physical and cognitive dimensions explained ‎‎0.23% of the variance of generalized anxiety, with Beta coefficients of 0.21 and 0.3, ‎respectively (Table 4). ‎

In the next step, to test the mediating role of experiential avoidance and repetitive ‎thinking in the relationship between anxiety sensitivity and generalized anxiety, all ‎variables were entered into the regression equation. By entering experiential avoidance ‎and repetitive thinking with anxiety sensitivity factors simultaneously, the cognitive ‎factor of anxiety sensitivity did not significantly explain generalized anxiety anymore, ‎but repetitive thinking (β = 0.32) and experiential avoidance (β = 0.22), significantly ‎explained this variable(Table 5). Based on this finding, we can infer that repetitive thinking and ‎experiential avoidance completely mediate the relationship between the cognitive ‎factor of anxiety sensitivity and generalized anxiety (Figure 1).‎

## Discussion

The aim of this study was to examine the role of mediating variables in the relationship ‎between anxiety sensitivity and generalized anxiety. In this study, a model was ‎examined in which anxiety sensitivity was a high-level factor, and experiential ‎avoidance and repetitive thinking were second level factors, and generalized anxiety ‎the outcome variable. The study findings could integrate and extend previous findings ‎by presenting a consistent pattern. The first hypothesis was that anxiety sensitivity ‎predicts experiential avoidance and repetitive thinking. This finding puts this study ‎among the studies emphasizing anxiety sensitivity as a fundamental element in anxiety ‎disorders. Anxiety sensitivity is a variable that affects the cognitive evaluation system; ‎and therefore, causes the person to lose his sense of control over situations and to ‎consider life events as potentially harmful (11, 24). Many studies indicate that in ‎addition to anxiety sensitivity, worry plays an important role in the pathology of ‎anxiety disorders. Therefore, in this study we aimed to take a wider perspective and ‎instead of limiting worry to contents, consider it as a trans-diagnostic factor. Thus, ‎instead of using worry or rumination scales, we used the Perseverative Thinking ‎Questionnaire (PTQ), which assesses the repetitive thinking process instead of contents. ‎According to the results of correlations and multiple regression analysis, it seems that ‎anxiety sensitivity predicts this process, except for the dimension of worry about ‎physical symptoms. The dimension of social worries is also associated with repetitive ‎thinking, a finding consistent with the previous studies on social anxiety (19, 25 and 26). ‎However, more studies are needed to examine the mediating role of repetitive thinking ‎in the relationship between the dimension of social worries and social anxiety. ‎Nevertheless, in line with previous studies and theories (13), the dimension of social ‎worries is most correlated with repetitive thinking. It seems that when people with ‎generalized anxiety face anxiety symptoms and negative predictions of events, they use ‎rumination and worry to maintain control over their cognitive processes, and even ‎though they relatively have positive beliefs about this, if they feel a lack of control over ‎their cognitive processes, it makes them vulnerable to generalized anxiety (27).‎

Moreover, consistent with the previous studies, a relationship was found between the ‎total score and anxiety sensitivity factors with experiential avoidance (28). However, in ‎contrast to the findings of this study, many previous studies, like Picket et al‏ ‏‎(2012) ‎have shown that experiential avoidance predicts anxiety sensitivity (29), but according ‎to the model described by Frank and Davidson (2014), it appears that anxiety ‎sensitivity acts as a predisposing trans-diagnostic factor, and experiential avoidance as a ‎reactive trans-diagnostic factor (30). In addition, according to the cognitive behavioral ‎model of emotional disorders, thoughts and beliefs lead to using behavioral strategies ‎‎(e.g., avoidance, reassurance seeking, checking, etc.) (31). Therefore, because people ‎with generalized anxiety believe that anxiety leads to negative physical and cognitive ‎consequences, they try to avoid anxiety-provoking situations. According to the ‎Borkovec's avoidance model of worry and anxiety (32), In addition to behavioral and ‎assurance seeking, worry is a cognitive avoidance mechanism that prevents people from ‎facing mental, physical, and emotional aspects of anxiety. The intolerance of ‎uncertainty model (33) maintains that worry as an effort to avoid uncertainty is ‎negatively reinforced, and prevents a change in a person’s beliefs about threat. ‎Therefore, repetitive thinking as an avoidance mechanism and an impaired cognitive ‎process, which can result from anxiety sensitivity, intolerance of uncertainty, and ‎maladaptive metacognitions leads to maintenance and intensification of anxiety ‎symptoms. This study was the first to simultaneously examine anxiety sensitivity, ‎experiential avoidance and repetitive thinking as a trans-diagnostic model of generalized ‎anxiety. Due to the fact that often co-morbidities exist between generalized anxiety ‎disorder and other emotional disorders such as major depression, panic, OCD, etc.(2), ‎the present model helps to explain co-morbidity and design a therapeutic protocol ‎based on trans-diagnostic factors. It also helps to compare the importance of variables in ‎predicting generalized anxiety severity, while previous studies have not examined it. ‎

## Limitations

The first limitation of this study was using a student, nonclinical sample; thus, ‎generalizing the findings to clinical or non-student groups should be done with caution. ‎It is also important to note that this study was conducted simultaneously with another ‎study, so the high number of questioners may have made the participants tired and less ‎motivated to answer the questions. Therefore, it is suggested that this study be ‎replicated in general and clinical populations, using survey and experimental methods ‎to make possible the generalizability of the data and to understand casual relationships.‎

## Conclusion

In summary, it seems that in unlike anxiety sensitivity, specially sensitivity to cognitive ‎impairment, people with GAD use worry to avoid this anxiety disadvantage, but this ‎makes the symptoms more severe, meaning that reducing anxiety sensitivity and ‎improving cognitive control may progress GAD treatment. Future studies can examine ‎the relationship between anxiety sensitivity and impaired cognitive control as well as ‎targeting anxiety sensitivity and impaired cognitive control to reduce GAD symptoms.‎
